# Epidemiological Survey on Porcine Cysticercosis in Nay Pyi Taw Area, Myanmar

**DOI:** 10.1155/2015/340828

**Published:** 2015-02-26

**Authors:** Tin Aye Khaing, Saw Bawm, Soe Soe Wai, Ye Htut, Lat Lat Htun

**Affiliations:** ^1^Department of Veterinary and Slaughterhouses, Nay Pyi Taw Development Committee, Nay Pyi Taw, Myanmar; ^2^Department of Pharmacology and Parasitology, University of Veterinary Science, Nay Pyi Taw 15013, Myanmar

## Abstract

Cross-sectional surveys were conducted to determine the prevalence and associated risk factors of *Taenia solium* cysticercosis in pigs within Nay Pyi Taw area, Myanmar. Meat inspection in three slaughterhouses, ELISA test, and questionnaire surveys were conducted in this study. Three hundred pigs were inspected in slaughterhouses and 364 pigs were randomly selected and examined from 203 households from three townships in Nay Pyi Taw area. The prevalence of porcine cysticercosis in meat inspection was 23.67% (71/300). Seroprevalence of *T. solium* cysticercosis in pigs in the study area was 15.93% (58/364). Significant associated risk factors with *T. solium* cysticercosis were gender (OR = 3.0; 95% CI = 1.7–5.4), increased age (OR = 2.3; 95% CI = 1.2–4.2), husbandry system (OR = 5.1; 95% CI = 2.4–11.2), feed type (OR = 16.9; 95% CI = 2.3–124.3), not using anthelmintics in pigs (OR = 11.9; 95% CI = 5.0–28.5), not using anthelmintics in owner (OR = 2.5; 95% CI = 1.4–4.4), no hand-washing before feeding (OR = 31.5; 95% CI = 4.3–230.9), and pork consumption of owner (OR = 37.4; 95% CI = 9.0–156.1) in the study area. This is the first report of porcine cysticercosis in Myanmar.

## 1. Introduction

Human neurocysticercosis (NCC) is caused by larval stage of zoonotic tapeworm* Taenia solium* (pork tapeworm) which remains a major public health problem in developing and some developed countries [1]. The World Health Organization estimates that eight people per 1000 worldwide have NCC [2]. This leads to epilepsy, madness, blindness, and death [3]. NCC can also occur in individuals who do not raise pigs or consume pork. Porcine cysticercosis is the cause of human taeniasis and neurocysticercosis is a consequence of taeniasis. Based on the available information, a very conservative and rough economic estimate indicates that the annual losses due to porcine cysticercosis in 10 west and central African countries amount to about 25 million Euros [4]. Ito et al. [5] also stated that, in China, the amount of pork discarded in the whole country due to cysticercosis annually has been estimated as 200,000,000 kg with a value of more than US $120,000,000. Dorny et al. [6] stated that notably data on Myanmar are lacking, although there are several reports of porcine cysticercosis based on meat inspection in the abattoirs in neighboring countries, 9.3% in India [7], 32.5% in Nepal [8], 5.4% in China [9], 0.02–2.63% in Indonesia [10], and 0.04 to 0.9% in Vietnam [6].

Although most of Myanmar culinary habits are based on thorough cooking, new food style such as barbecue and dishes based on raw or undercooked pork or pork product becomes popular among customers. Moreover, small-scale pig husbandry has become one of the major sources of income in Myanmar farmers. So it may be high risk of getting food-borne zoonotic diseases according to the new food style and traditional husbandry method. Due to lacking of information on porcine cysticercosis in Myanmar up to now, it is important to investigate the prevalence and associated risk factors.

Nay Pyi Taw area, the capital of Myanmar, has big population of pigs (about 200,000 pigs) [11] to support the demand of pork consumption in this area. Most of the pig farmers are smallholders and most of pig husbandry systems are free ranging or semi-intensive with lack of proper sanitation.

One of the main obstacles to control the* T. solium* infections is the lack of adequate epidemiological data on cysticercosis/taeniasis. Therefore, the objectives of this community-based study were to investigate the prevalence of porcine cysticercosis and associated risk factors in pigs within study area. Moreover, findings of this study will assist to develop the control strategies of porcine cysticercosis for the public health aspect.

## 2. Materials and Methods

### 2.1. Study Design, Study Area, and Sample Size

The cross-sectional studies were conducted from January to March and June to July 2014, to investigate the prevalence of* Taenia solium* cysticercosis in slaughtered and farmed pigs within Pyinmana, Lewe, and Tatkon townships, Nay Pyi Taw area. It is located between latitude 19°45′N and longitude 96°6′E and with climate data; the altitude is 115 m above sea level, annual rainfall is 115 mm, and annual temperature is 21.2–32.5°C. The targeted population was 180,000 pigs in three townships [11] during the sampling period. The number of sampled pigs was calculated using the formula stated by Thrusfield [12]. An expected prevalence of 30% with a confidence level of 95% was used in this unit. In this study, 300 slaughtered pigs and 364 farmed pigs from the study area were examined although calculated samples were 298 and 323, respectively (Table 1). Blood collected from the jugular vein of farmed pig was conducted for the seroprevalence and a structured questionnaire with both closed and open-ended questions was administered to owners to obtain management practices in pig husbandry. Piglets younger than two months, pregnant sows, and nursing sows with litters less than two months old were excluded from this study to overcome the stress which causes adverse effect in animals.

### 2.2. Meat Inspection in Slaughterhouses

Meat inspection was carried out as described by Boa et al. [13] in the three slaughterhouses of these townships. There were 300 randomly selected pigs recruited and 9 different muscles (tongue, masseter, brain, shoulder, diaphragmatic, heart, skeletal, fore limb, and hind limb muscle) from each pig in meat inspection. Briefly, long and parallel incision into the masseter muscles on both sides of face in an upward direction was made. A deep longitudinal incision covering about 3/4 the thickness of the tongue and covering the whole length of the tongue was made to examine the cysts. After opening the pericardium, the heart was also visually examined for the presence of cysts. The heart was cut open and a deep (3/4 the thickness of septum) incision into the septum was made to expose any metacestodes. All the other muscles were viewed, palpated, incised by surgical blade, and visually examined.

### 2.3. Blood Collection and Antibody-ELISA Test for the Detection of IgG Antibody of* T. solium* Cysticerci

The pig was kept under restraint at standing position and blood samples were obtained from the external jugular vein by using sterile disposable syringes and put into vacutainers with clot activators. Those vacutainers were kept in cold boxes with ice and transported to Department of Pharmacology and Parasitology, University of Veterinary Science, Nay Pyi Taw, and allowed overnight at 4°C to clot. To obtain serum, the clotted blood was separated by centrifugation at 4000 rpm for 10 minutes. The clear sera were transferred to 1.5 mL microvial tubes and stored in labeled wails and kept at −20°C until analysis.

Detection of IgG antibody of* T. solium* cysticerci was carried out by using antibody-ELISA kit (NovaTec Immundiagnostica GMBH Co., Belgium) according to manufacturer's instruction. Briefly, all thawed samples were diluted as 1 + 100 with IgG Sample Diluent (phosphate buffer) before assaying. The 100 *μ*L controls and diluted samples were dispensed into their respective wells and the foil was covered. After incubation for 1 hour at 37°C and the foil being removed, the contents of the wells were aspirated and washed three times with washing solution. And then, remaining fluid was carefully removed by tapping strips on tissue paper. The 100 *μ*L protein A conjugate (horseradish peroxidase) was dispensed into all wells except A1 and covered with foil and incubated for 30 min at room temperature. After washing three times, 100 *μ*L TMB (3,3′,5,5′-tetramethylbenzidine) substrate solution was dispensed into all wells and incubated for exactly 15 min at room temperature in the dark. The reaction was stopped by adding 100 *μ*L stop solution (0.2 M H_2_SO_4_). The absorbance was determined at 450/620 nm using an ELISA reader (Stat Fax). In each ELISA kit testing, there are two cut-off controls (C_1_ and D_1_). The mean absorbance of these cut-off controls was used as cut-off value. Samples are considered “positive” if the absorbance value is higher than 10% over the cut-off and samples are considered “negative” if the absorbance value is lower than 10% below the cut-off. The sensitivity and specificity of these kits to diagnose swine cysticercosis are 93.8% and >95%, respectively.

### 2.4. Household Questionnaire

A questionnaire was developed and used to collect information on hypothesized risk factors and other related pieces of information from sampled pig owners. Households in each township were selected by using the snowballing technique from those farmers willing to participate in the study. It is a technique for developing a research sample where existing study subjects recruit future subjects from their acquaintances.

### 2.5. Statistical Analysis

The questionnaire interviewed data were analyzed for the relationship between the prevalence of* T. solium* cysticercosis and hypothesized risk variables such as age, gender of pigs, husbandry system, feed type, environment of pig farm (accessibility of human feces), personal hygiene of owners, pork consumption, cooking and eating habit of pork, use of anthelmintics in pigs and owners, and knowledge on taeniasis. They were examined for testing its significance by Pearson chi-square test at *α* = 0.05. The data were analyzed by using SPSS (version 16).

## 3. Results

### 3.1. Seroprevalence of Porcine Cysticercosis in Farmed Pigs

Seroprevalence of porcine cysticercosis in farmed pigs was 15.93% (58/364) in the study area.

### 3.2. Prevalence of Households with Porcine Cysticercosis

Prevalence of households with pigs infected with* T. solium* cysticerci by Ab-ELISA examination was 23.15% (47/203 households). The households with porcine cysticercosis in Pyinmana, Lewe, and Tatkon were 0/12 (0%), 13/124 (10.48%), and 34/67 (50.75%), respectively.

### 3.3. Prevalence of Porcine Cysticercosis in Meat Inspection

The prevalence of porcine cysticercosis investigated by meat inspection was 23.67% (71/300). All the infected pigs presented parasites located in the tongue. Only in one pig, another parasite was found, located in the heart. In none of the animals evaluated, parasites were found in the other locations examined. The prevalence in slaughterhouses of Pyinmana, Lewe, and Tatkon townships was 22% (44/200), 23.33% (7/30), and 28.57% (20/70), respectively.

### 3.4. Risk Factors Associated with Porcine Cysticercosis

Univariate analysis of hypothesized risk factors of gender (OR = 3.0; 95% CI = 1.7–5.4), increased age (OR = 2.3; 95% CI = 1.2–4.2), husbandry system (OR = 5.1; 95% CI = 2.4–11.2), feed type (OR = 16.9; 95% CI = 2.3–124.3), no hand washing habit before feeding (OR = 31.5; 95% CI = 4.3–230.9), not using anthelmintic in pigs (OR = 11.9; 95% CI = 5.0–28.5) and owner (OR = 2.5; 95% CI = 1.4–4.4), and pork consumption of owner (OR = 37.4; 95% CI = 9.0–156.1) was significantly associated with* Cysticercus cellulosae* infection (*P* < 0.05). The distribution and odds ratio of significant risk factors concerning porcine cysticercosis are shown in Table 2.

## 4. Discussion

In Southeast Asia, pigs are an important source of food and economic important for smallholder farmers. Older pigs may be penned or tethered although common raising practice of pigs is freely roaming in the village [14]. In Myanmar, most of the pig farmers are smallholders and practice as free-range or backyard farming. In Myanmar, most of the pig farmers usually keep the weaned pigs until six to eight months of age and then send to slaughterhouse. In the village, every household keeps at least one pig not only for table waste feeding to pigs but also for extra income. Most farms are having the habit of feeding waste materials such as swill and kitchen leftover, broken rice, rice bran, groundnut meal, sesame meal and local forage, and poor sanitation.

The present study is the first report of* T. solium* cysticercosis in pigs in Myanmar. This investigation showed relatively high prevalence of porcine cysticercosis in the study area. Pigs in the study area positive for cysticercosis have been exposed to* T. solium *eggs. Among the 17 hypothesized risk factors, eight factors were evaluated as having association.

The gender of pigs (being female) was significantly associated with porcine cysticercosis in this study. It can be explained that female pigs were for kept long time for breeding purpose than male and so they have more risk to get exposed to* T. solium* eggs. However, Jayashi et al. [15] reported that gender was not a significant risk factor for porcine cysticercosis.

The present study demonstrated that the older the pigs, the greater the chance to get infection. These results are in agreement with those reported by Pouedet et al. [16], Jayashi et al. [15], Sarti et al. [17], García et al. [18], and Pondja et al. [19]. Older pigs might also have greater chance to get exposed to* T. solium* eggs than younger ones. They might have much time to develop cyst and trigger the production of circulating antibodies. Besides, it could be possible that younger pigs are protected during their first months of life against parasite infection, due to the presence of maternal cysticercus antibodies and they become susceptible later after the slow clearance of those antibodies.

The result showed that pigs from households practiced semi-intensive system (the pigs are allowed to roam freely in the environment and only panned or tethered at feeding time and night) were more likely to have porcine cysticercosis than intensive (the pigs are kept in the backyard or corral and not allowed to roam) pigs. Therefore, semi-intensive management system represented as an important risk factor for porcine cysticercosis in the study area as the pigs in this practice could access the infected human faeces. Accessibility of infected human faeces is the main source for porcine cysticercosis [17, 19, 20].

Among the feed types used in pig farms, feeding of kitchen waste is significantly associated with cysticercosis. In the farms, most of housewives usually collect swill in poor cleanliness containers from neighboring houses. This might be contaminated with* T. solium* eggs from infected food preparers of swill collected houses. So the collected swill should be cooked thoroughly before feeding to prevent infection including cysticercosis. Human taeniasis is the main source for porcine cysticercosis [21].

Use of anthelmintic in pigs and owners was significantly associated in this study. By interviewing the farmers and township veterinary officers, the most common used anthelmintic is albendazole in human and ivermectin in pigs. Although ivermectin cannot kill any larvae of cestode, albendazole can kill these larvae. Not having taeniasis in owners is preventive factor against cysticercosis [18].

Although all the farmers wash their hands after feeding the pigs, only 21.2% famers (43/203) wash their hands before feeding. All cysticercosis positive samples were from those who do not practice hand-washing habit. Therefore, hand-washing is a crucial factor for prevention of porcine cysticercosis. However, there was no literature about this factor associated with porcine cysticercosis. But health education and sanitary infrastructure are involved in the control measure for swine cysticercosis [17].

About half of farmers (114/203) consume the pork curry in this study. All positive samples were from the owners consumed pork (51 households). They might have taeniasis and cysticercosis due to cooking habit and poor sanitation. Pork consumption of owners is also one of the risk factors in survey of porcine cysticercosis [19].

Nine hypothesized risk factors not included in analysis were breed of pigs, place of purchase, presence of latrine, hand-washing after feeding the pigs, source of water for pigs, cleanliness of water, knowledge on taeniasis and cysticercosis, and occurrence of cyst in pork. In this study, all pigs are indigenously bred. All pigs were purchased from within their township. All farmers have latrines using water, but the children do not use latrine and are used for defecation out of latrine. Some farmers washed the hands before feeding the pigs and all farmers washed their hands after feeding. All farmers used water from wells having good sanitation. All farmers did not have the knowledge on taeniasis and cysticercosis and they have never seen the cysts in the pork in the study area.

The presence of zoonotic agent,* Cysticercus cellulosae*, may depend on intrinsic factors: age, gender, and extrinsic factors: pig husbandry system, hand-washing habit of owner, use of kitchen waste as pig feed, not using anthelmintic in pigs and owners, and pork consumption of owner in the study area. Presence of this infection is of public health importance because it may lead to the occurrence of neurocysticercosis in human.

Although the occurrence of human neurocysticercosis has not been reported yet in Myanmar, all public should take awareness of potential risk factors due to the prevalence with high percentage observed in this study. Myanmar has no national monitoring program for* T. solium* cysticercus spp. in these animals yet. Therefore, it is advisable to monitor whether there is high or low prevalence of* T. solium* cysticercosis in the whole country. It could also be suggested that confinement housing system should be developed in pig industry of Myanmar to efficiently prevent porcine cysticercosis. For practicing sanitary and culinary habit, thorough cooking education programs should also be implemented for both swine breeders and consumers so as to prevent taeniasis in human and porcine cysticercosis and also other zoonotic helminth diseases in Myanmar.

This prevalence with relatively high percentage of porcine cysticercosis (15.93%) in Ab-ELISA and 23.67% in slaughtered pigs indicates the presence of human taeniasis and it also leads to the associated risk of human cysticercosis and neurocysticercosis. Occurrence of porcine cysticercosis poses a serious problem to human health and the economy. Therefore, it is required to monitor porcine cysticercosis prevalence in the whole country.

## Figures and Tables

**Table 1:** Distribution of the number of samples in pigs within the three townships for blood collection.

Number	Township	Pig population	Number of sampled pigs
1	Pyinmana	39,000	81
2	Lewe	86,000	172
3	Tatkon	55,000	111

	Total	180,000	364

**Table 2:** Distribution and odds ratio of associated risk factors concerning porcine cysticercosis in Nay Pyi Taw area.

Factor	Level	*n*	Positive case	Negative case	Odds ratio	*P* value
Gender	Male	221	22	199	3.043 (1.704–5.436)	0.000^*^
	Female	143	36	107		

Age	<6 month	291	39	252	2.274 (1.220–4.235)	0.012^*^
	≥6 month	73	19	54		

Husbandry system	Intensive	146	8	138	5.134 (2.354–11.195)	0.000^*^
	Semi-intensive	218	50	168		

Feed type	Only commercial feed	71	1	70	16.907 (2.300–124.302)	0.000^*^
	Both with kitchen waste	293	57	236		

Use of anthelmintic in pigs	Yes	183	6	177	11.891 (4.957–28.526)	0.000^*^
	No	181	52	129		

Use of anthelmintic in owner	Yes	242	28	214	2.492 (1.409–4.407)	0.002^*^
	No	122	30	92		

Hand-washing before feeding	Yes	110	1	119	31.538 (4.307–230.92)	0.000^*^
	No	254	57	197		

Pork consumption of owner	No	177	2	175	37.405 (8.965–156.068)	0.000^*^
	Yes	187	56	131		

^*^Significant association at 0.05 level.
